# A Low-Noise MEMS Accelerometer Based on a Symmetrical Sandwich Capacitor Structure

**DOI:** 10.3390/mi17020271

**Published:** 2026-02-22

**Authors:** Zihan Wang, Chaowei Si, Jihua Zhang, Zhen Fang, Jinxu Liu, Shuqi Li, Wanli Zhang

**Affiliations:** 1 School of Integrated Circuit Science and Engineering, University of Electronic Science and Technology of China, Chengdu 610054, China; 18833290514@163.com (Z.W.);; 2 State Key Laboratory of Electronic Thin Films and Integrated Devices, University of Electronic Science and Technology of China, Chengdu 610054, China; 3 Institute of Semiconductors, Chinese Academy of Sciences, Beijing 100083, China

**Keywords:** MEMS accelerometer, anodic bonding, low noise

## Abstract

This study presents a high-performance MEMS accelerometer employing a symmetrical differential ‘sandwich’ capacitive structure. An orthogonal rectangular compensation method was integrated with wet anisotropic etching to achieve high structural symmetry. An innovative glass–silicon composite cover plate was adopted, and the upper and lower plates were encapsulated by a sensitive structure via anodic bonding, which effectively reduced the parasitic capacitance. Simulations confirmed sufficient separation between the sensitive-axis (Z-axis) resonant frequency and orthogonal/torsional modes, ensuring low cross-axis coupling. The fabricated device exhibits high sensitivity (0.2216 V/g) and excellent linearity (99.842%) within a 0–8 g range. Furthermore, it demonstrates outstanding noise (7.88 µg/√Hz) and bias-instability (6.39 µg) performance, positioning it competitively against commercial counterparts. The proposed design and process offer a viable technical route for high-precision inertial sensing applications.

## 1. Introduction

The MEMS accelerometer is a miniaturized sensor manufactured via micro-electro-mechanical system (MEMS) technology. Its operational principle is based on the capacitive sensing of minute deflections in an internal spring-mass (proof mass) structure subjected to inertial forces. These deflections, which are proportional to the applied linear acceleration (including gravity), are transduced into measurable changes in capacitance [1,2,3]. Characterized by miniaturization, low cost, low power draw, and robustness, this sensor is ubiquitously deployed in applications from mobile phone screen rotation and automotive airbags to industrial vibration monitoring, serving as a fundamental motion-perception unit in smart devices [4,5].

The “sandwich” capacitive MEMS accelerometer features a vertically stacked architecture with a movable proof mass sandwiched between two fixed electrodes, forming a differential capacitive sensing unit. Acceleration causes the proof mass to deflect, differentially altering the gaps to the electrodes and generating opposing capacitance changes. This capacitive imbalance is measured through a differential readout to determine the acceleration’s magnitude and direction [6,7,8]. This symmetrical differential design confers several key performance advantages. First, it enhances sensitivity and the signal-to-noise ratio by providing a differential output that amplifies the signal while inherently suppressing common-mode interference. Second, it offers improved linearity, reduced cross-axis sensitivity, and greater robustness against overload, the latter aided by integrated mechanical stoppers. Typically fabricated via advanced processes like glass–silicon–glass bonding or silicon-on-insulator (SOI) micromachining, these devices achieve a hermetic seal and precise gap control [9,10,11]. Consequently, this high-performance accelerometer has become the preferred solution for demanding applications in automotive electronics (e.g., ESP, airbags), industrial automation (e.g., platform stabilization, tilt sensing), inertial navigation, and high-end consumer devices [12,13].

In this work, a symmetric sandwich-style MEMS accelerometer was fabricated. Structural symmetry was achieved via wet etching, while device encapsulation and parasitic capacitance reduction were accomplished using a glass-in-silicon (GIS) reflow process. The resulting device subsequently exhibits a high dynamic range, low nonlinearity, and low noise characteristics.

## 2. Design and Manufacture of Accelerometer

The working principle of MEMS accelerometer is based on Newton’s second law, which converts acceleration measurement into sensitivity. The displacement and mechanical properties of the mass block are measured to obtain the output value of the acceleration. According to Newton’s second law, when a sensitive mass is subjected to an external force and the acceleration is a, the acceleration is

(1) $$m\ddot{x}+d\dot{x}+kx=ma$$

where *m* is the mass of the sensitive mass, x is the displacement of the mass, and d is the equivalent damping system of the mass. k is the equivalent stiffness of the system. Equation (1) performs Laplace transform on the initial state:

(2) $$\left(ms^{2}+ds+k\right)X\left(s\right)=mA\left(s\right)$$

The transfer function of the system can be obtained by simplifying the ratio of the output response displacement to the input acceleration [14]:

(3) $$H\left(s\right)=\frac{X\left(s\right)}{A\left(s\right)}=\frac{1}{s^{2}+\frac{\omega }{Q}s+\omega^{2}}$$

(4) $$\omega =\sqrt{\frac{k}{m}}$$

(5) $$Q=\frac{\sqrt{km}}{d}$$

where ω is the intrinsic resonant frequency of the system and Q is the quality factor of the system.

For MEMS accelerometers, where the natural frequency *ω*_0_ is significantly lower than the cut-off frequency, *ω*_c_, within the operating band, the viscous damping coefficient approximates a constant c, with negligible elastic damping. Thus, the amplitude-frequency response simplifies to

(6) $$\omega_{c}=\frac{\pi^{2}h_{0}^{2}P_{a}}{12\mu \omega^{2}}$$

The amplitude-frequency response of the system is

(7) $$A_{0}=\frac{F_{0}}{m}\sqrt{\frac{1}{{\left(\omega_{0}^{2}-\omega^{2}\right)}^{2}+4\xi^{2}\omega_{0}^{2}\omega^{2}}}$$

When *ξ* < 1, the system is underdamped; when *ξ* = 1, it is critically damped; and when *ξ* > 1, it becomes overdamped. The accelerometer designed in this work is a high-sensitivity device, which requires the sensitive structure to be maintained in an overdamped state.

The noise of the pendulum MEMS accelerometer mainly includes the mechanical noise and the circuit noise. The mechanical noise is mainly Brownian noise. Total noise density equivalent acceleration is abbreviated as *TNEA* (total noise density). The expression of the equivalent acceleration is [15]

(8) $$TNEA=\sqrt{BNEA^{2}+CNEA^{2}}\left[\frac{{m/s}^{2}}{\sqrt{Hz}}\right]$$

where *CNEA* is the noise of the detection circuit, and *BNEA* is the Brownian noise equivalent acceleration of the accelerometer.

(9) $$BNEA=\sqrt{\frac{4k_{B}T\omega_{0}}{MQ}}\left[\frac{{m/s}^{2}}{\surd Hz}\right]=\frac{1}{g}\sqrt{\frac{4k_{B}T\omega_{0}}{MQ}}\left[g/\surd Hz\right]$$

where $k_{B}$ is the Boltzmann constant, *T* is the temperature, $\omega_{0}$ is the resonant frequency of the sensitive structure, and *M* is the mass of the mass block.

**Figure 1:**
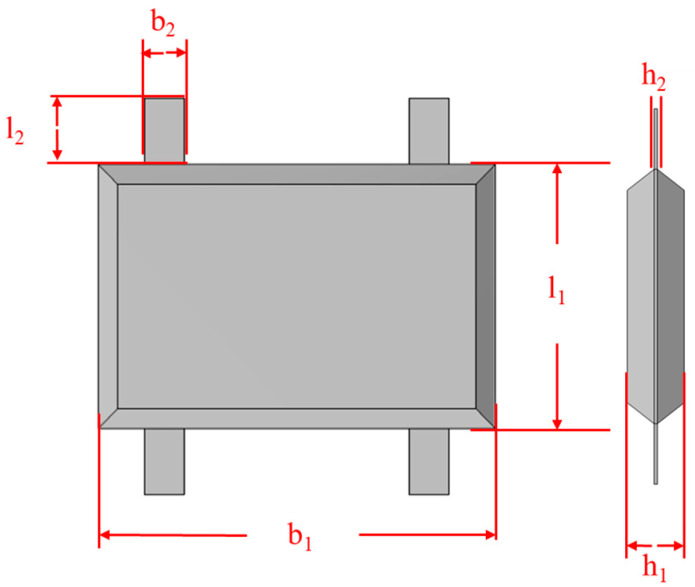
Accelerometer sensitive structure and key parameters schematic.

The core sensitive structure of the accelerometer is composed of a mass block and an elastic beam supporting the mass block (as shown in Figure 1). The sensitive structure contains seven key parameters, where b_1_, l_1_, and h_1_ are the length, width, and thickness of the mass; l_2_, b_2_, and h_2_ are the length, width, and thickness of the flexible beam; and Gap is the initial gap between the mass and the electrode plate. The specific parameters are shown in Table 1.

The geometric model of the pendulum-sensitive structure was constructed in the COMSOL Multiphysics 6.1 simulation software. In this model, the sensitive proof mass is suspended and connected to a frame by four elastic beams; their key dimensions are listed in Table 1. Finite element analysis (FEA) was subsequently performed to determine the resonant frequencies of the structure along the X-, Y-, and Z-axes, with the Z-axis defined as the sensitive direction of the device.

**Figure 2:**
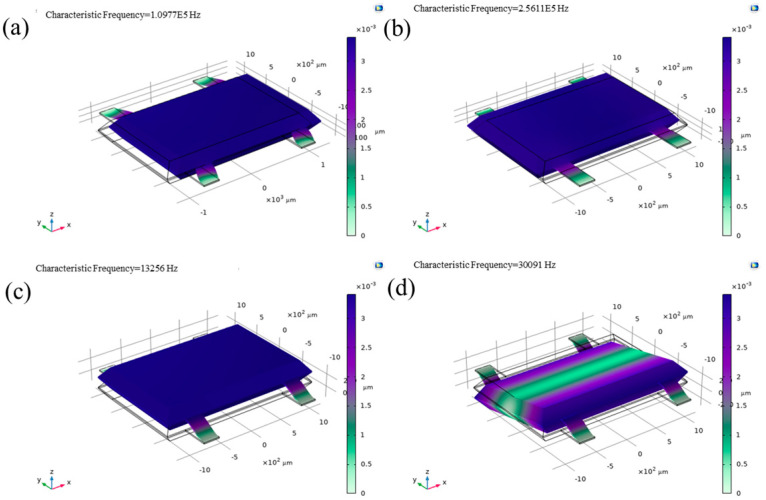
Sensitive structure resonance mode simulation (**a**) X-axis, (**b**) Y-axis, (**c**) Z-axis, (**d**) out-of-plane torsion mode.

**Figure 3:**
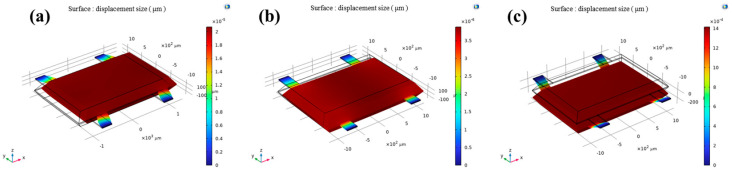
Total displacement with 1 g acceleration in (**a**) Z-axis, (**b**) X-axis, (**c**) Y-axis.

The finite element simulation results (Figure 2) show that the structure’s first resonant mode, corresponding to the sensitive Z-axis direction, occurs at 13,256 Hz. This is distinctively lower than the resonant frequencies in the X-axis (109,772 Hz) and Y-axis (256,110 Hz) directions, as well as the out-of-plane torsional mode (30,091 Hz). Figure 3 shows the displacements of the three axes at 1 g acceleration. This frequency separation is critical for operational stability. Based on Equation (4), the calculated stiffness (Table 2) in the Z-axis is substantially lower than that in the orthogonal X- and Y-axes. Consequently, this anisotropic stiffness design inherently suppresses mechanical cross-coupling for accelerations applied off the sensitive axis.

**Figure 4:**
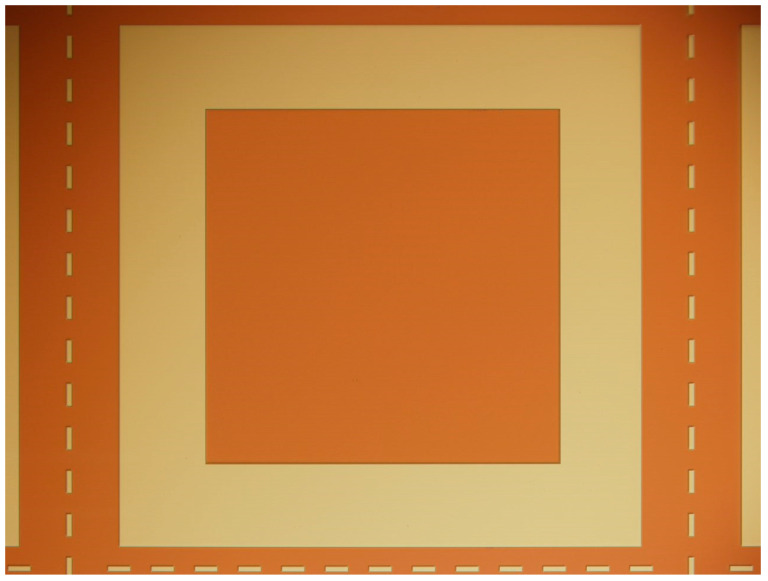
Accelerometer cover plate.

The pendulum-sensitive structure was fabricated from a double-side-polished silicon wafer using anisotropic wet etching with tetramethyl ammonium hydroxide (TMAH). First, cavities defining the cap spacings were etched on both sides of the wafer. Subsequently, the sensitive proof mass and the elastic supporting beams were released through wet etching processes. A silicon–glass composite cap was prepared separately: its stepped structure was formed by deep silicon etching, followed by anodic bonding to a glass wafer. A glass reflow process was then employed to fill the silicon grooves, after which grinding and chemical-mechanical polishing (CMP) were used to re-expose the silicon surface. Finally, patterned metal electrodes were deposited via magnetron sputtering (As shown in Figure 4). The fabricated glass–silicon composite cover plate exhibits a strong capability for parasitic capacitance reduction [16].

The anisotropic wet etching of silicon in TMAH results in the undercutting of convex corners due to the faster etch rate of certain crystal planes. When fabricating the proof mass of a MEMS accelerometer, such undercutting can critically distort the structure [17]. The standard mitigation strategy is convex corner compensation, where auxiliary geometric features (such as squares or bars) are added at the corner in the mask layout. These features alter the local etch front progression, causing the etchant to attack the compensation pattern first, thus protecting the integrity of the actual device corner. Common compensation designs for (100)-silicon include squares, superimposed squares, directional bars, and orthogonal rectangles [18].

**Figure 5:**
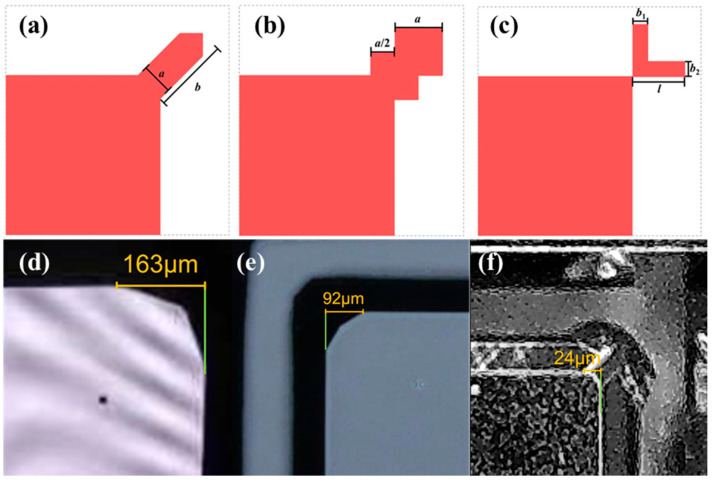
The effects of different convex angle compensation schemes: (**a**) strip compensation, (**b**) superimposed square compensation, (**c**) orthogonal rectangular compensation, (**d**–**f**) is the corresponding physical map after corrosion.

As shown in Figure 5, the convex cutting sizes of the three methods are 163 μm, 92 μm and 24 μm, respectively. Orthogonal rectangular compensation can significantly suppress the convex cutting of the mass block.

**Figure 6:**
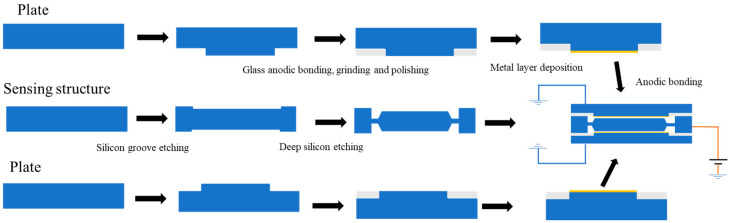
Device preparation process flow.

Following the separate fabrication of the pendulum-sensitive structure layer and the silicon–glass composite caps, the device was assembled. The upper and lower composite caps were precisely aligned with the central sensitive structure layer. Subsequently, these three layers were permanently bonded using an anodic bonding process. This forms the sealed, differential capacitive sensing cell and completes the three-dimensional packaging of the accelerometer. The overall process flow is illustrated in Figure 6.

**Figure 7:**
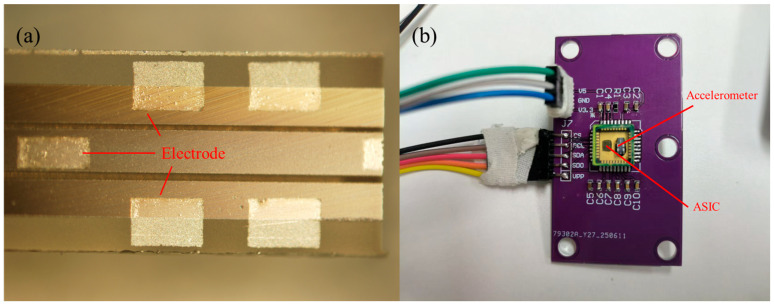
(**a**) Finished accelerometer product top view and electrode structure, (**b**) finished accelerometer device and test board.

The final accelerometer device and the ASIC chip of the detection circuit are packaged in the shell to form the accelerometer chip (as shown in Figure 7).

## 3. Results and Discussion

**Figure 8:**
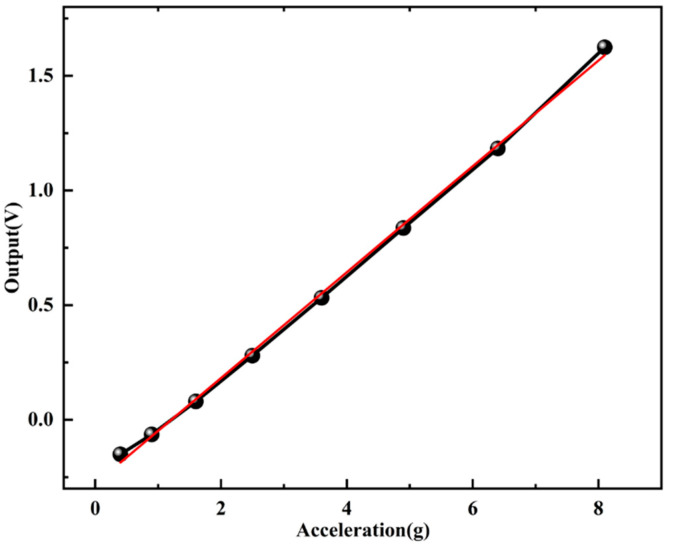
The test result of the scale factor.

The output response of the accelerometer was tested at room temperature (22 °C), and the sensitivity curves are shown in Figure 8. It can be seen that the output value of the accelerometer is linearly related to the input acceleration, and the sensitivity of the accelerometer is about 0.2216/g, indicating that the accelerometer designed in this paper can detect the acceleration in the direction of the sensitive axis (Z-axis) and has ultra-high sensitivity. In the range of 0~8 g, the linearity of the pendulum accelerometer is 99.842%, indicating that the pendulum accelerometer designed in this paper has very good linear output characteristics. On the one hand, this is due to the pendulum-sensitive structure design of the accelerometer. On the other hand, it is due to the use of a silicon–glass composite cover plate and silicon sensitive structure layer. This three-layer anodic bonding package greatly reduces the influence of parasitic capacitance on device performance.

**Figure 9:**
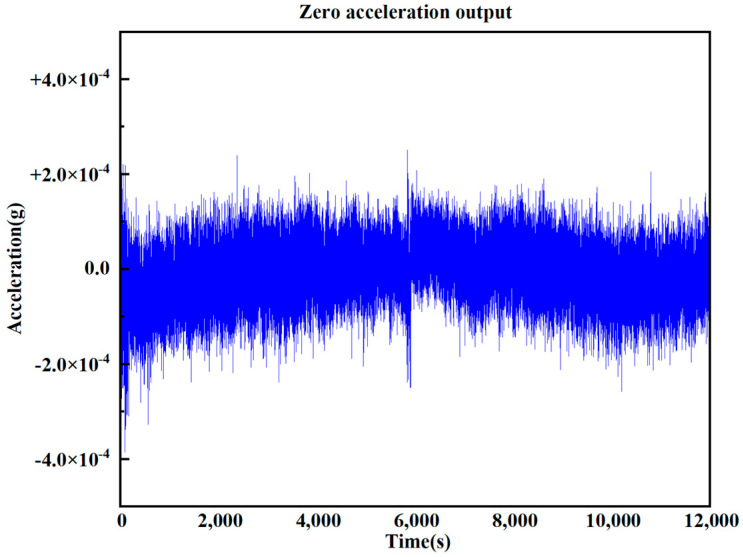
The zero-acceleration output.

The Zero-Rate Output (ZRO) is the output of an accelerometer without any acceleration input. It can reflect noise and output deviations in the accelerometer. The ZRO drift of the accelerometer is an important factor affecting its high-precision performance. The ZRO characteristics of the pendulum accelerometer were tested and characterized at room temperature (22 °C). The zero output superelevation of the device was continuously collected at a sampling frequency of 100 Hz for 3 h, and the ZRO test diagram of the accelerometer was obtained. As shown in Figure 9, the maximum ZRO drift is about 0.42 mg.

**Figure 10:**
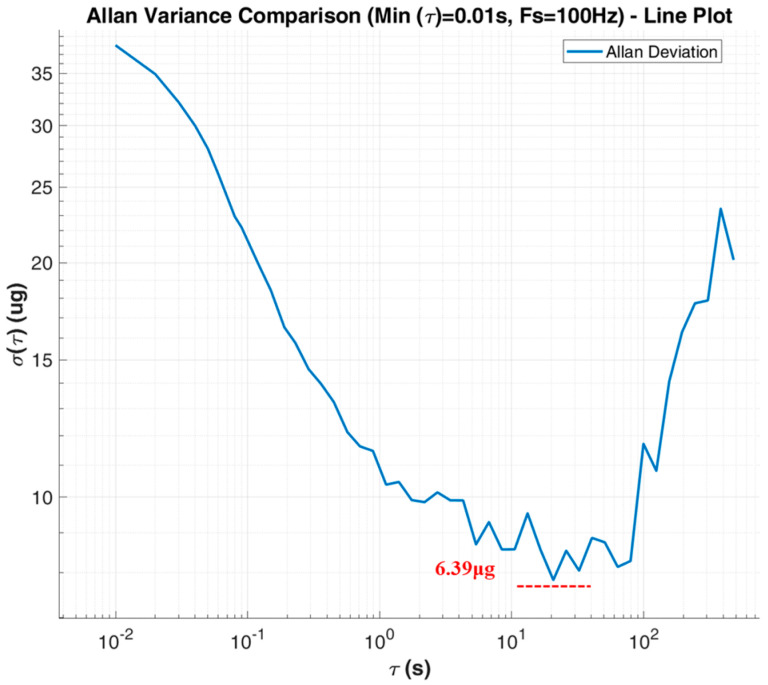
The Allan deviation.

**Figure 11:**
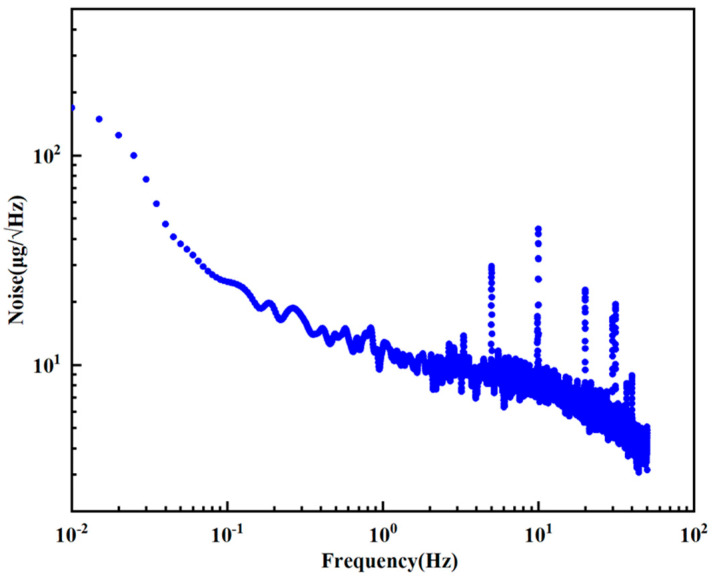
Noise power spectral density of accelerometers.

Bias instability reflects the offset characteristics of the accelerometer output over time without external acceleration input, which is usually characterized by the Allen variance or standard deviation of the ZRO curve. Allen variance analysis of the ZRO characteristic curve of the accelerometer at room temperature (22 °C) was performed to obtain an Allen variance characteristic diagram of the accelerometer. As shown in Figure 10, the bias instability of the accelerometer is 6.39 μg, indicating that the accelerometer designed in this paper has excellent comprehensive performance. The noise power density of the device was obtained by the Fourier transform of the ZRO of the device. As shown in Figure 11, the noise power density of the device is 7.88 μg/√Hz(@10HZ), indicating that the accelerometer designed in this paper has extremely low noise characteristics.

**Figure 12:**
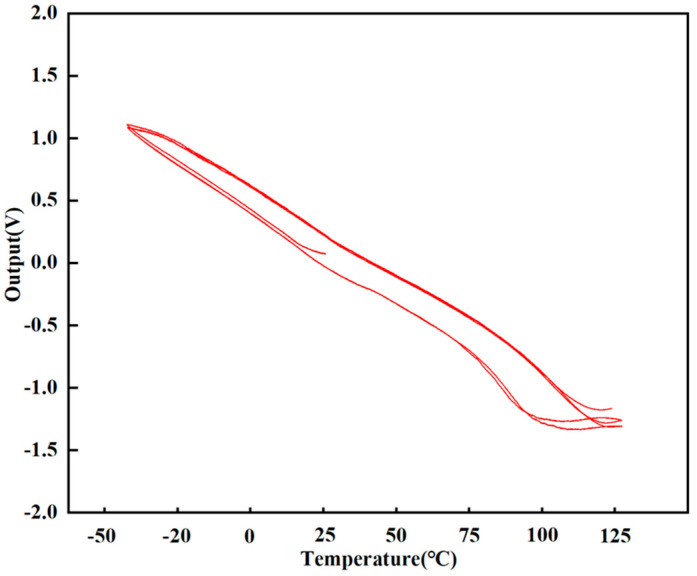
Accelerometer three temperature cycle zero output curve.

The device underwent two consecutive temperature cycling tests across a range of −40 °C to 125 °C. As shown in Figure 12, a minimal hysteresis was observed between the heating and cooling curves. Furthermore, the output exhibits excellent repeatability at identical temperature points across both cycles. These results demonstrate the device’s robust environmental stability and reliable performance under thermal stress.

Table 3 compares the performance of our device with pendulum accelerometers on the market. The accelerometer we prepared has low noise and low bias instability and is competitive.

## 4. Conclusions

In this study, a symmetrical differential capacitive MEMS accelerometer was successfully developed. A sensitive structure with high symmetry is realized by wet etching and a convex angle compensation process, and the parasitic capacitance was effectively reduced by combining a glass–silicon composite cover plate and three-layer anode bonding package. The simulation and test results show that the sensitive axis (Z-axis) of the device is well separated from the orthogonal axis modal frequency, and the cross-coupling is low. It has high linearity (99.842%) and high sensitivity (0.2216 V/g). At the same time, it has excellent low-noise characteristics (noise density 7.88 μg/√ Hz) and low bias instability (6.39 μg). The design and process scheme of the accelerometer provides an effective way to realize high-performance micro-inertial sensing, which is suitable for high-precision inertial navigation, platform stability and industrial monitoring.

## Figures and Tables

**Table 1:** Key parameters of the sensitive structure.

Parameters	Dimensions (μm)
b_l_	2400
l_1_	1600
b_2_	240
l_2_	400
h_1_	400
h_2_	20
Gap	3.2

**Table 2:** The resonant frequency of the finite element simulation and the corresponding stiffness and each axial displacement at 1 g.

	Resonance Frequency (Hz)	Equivalent Stiffness (N/m)	Unit Acceleration Displacement Response (m/g)
Z	13,256	456.8	2.04 × 10^−11^
X	109,772	31,329.7	3.69 × 10^−12^
Y	256,110	170,540.1	1.41 × 10^−9^

**Table 3:** Accelerometer performance comparison.

Manufacturer	Model	Noise (μg/√Hz)	Bias Residual (mg)
TDK (Tronics)	AXO315-T0	10	0.8
TDK (Tronics)	AXO314	4	1
MicroStrain (Lord)	G-Link-200-OEM	25	-
Colibrys	MS1002	7	-
Murata	SCA3-D3400	20	1.2
This work	-	7.88	0.42

## Data Availability

The original contributions presented in this study are included in the article. Further inquiries can be directed to the corresponding authors.
